# *EGFR*基因少见突变型非小细胞肺癌的临床特征及应用EGFR-TKIs治疗效果评价 

**DOI:** 10.3779/j.issn.1009-3419.2019.05.06

**Published:** 2019-05-20

**Authors:** 云舒 史, 盼华 李, 班班 李, 凤鸣 张, 思远 黄, 淑景 申, 醒亚 李

**Affiliations:** 1 450052 郑州，郑州大学第一附属医院肿瘤科 Department of Medical Oncology, the First Affiliated Hospital of Zhengzhou University, Zhengzhou 450052, China; 2 450052 郑州，郑州大学第一附属医院放疗科 Department of Tumor Radiotherapy, the First Affiliated Hospital of Zhengzhou University, Zhengzhou 450052, China

**Keywords:** 肺肿瘤, EGFR, 突变, 靶向治疗, EGFR-TKIs, Lung neoplasms, EGFR, Mutation, Target therapy, EGFR-TKIs

## Abstract

**背景与目的:**

晚肺腺癌是肺癌中最常见的类型，表皮生长因子受体酪氨酸激酶抑制剂（epidermal growth factor receptor tyrosine kinase inhibitors, EGFR-TKIs）目前已成为*EGFR*突变型非小细胞肺癌（non-small cell lung cancer, NSCLC）的一线标准治疗。经典型突变（19外显子缺失突变和21外显子L858R突变）应用EGFR-TKIs治疗的效果已有大量研究和报道，而少见或复合突变类型的相关报道较少，具体疗效尚未完全统一定论。

**方法:**

对2016年8月-2018年4月就诊于郑州大学第一附属医院的150例经基因检测证实为EGFR少见突变的NSCLC患者进行回顾性研究，分析其突变类型及临床病理特征，并对其中48例接受EGFR-TKIs Ⅰ/Ⅱ/Ⅲ线治疗的患者的疗效进行描述和评价。

**结果:**

将全部150例患者按突变类型分为4组，分别为18外显子G719X突变46例（30.7%）、21外显子L861Q突变45例（30.0%）、其他单一少见突变14例（9.3%）和复合突变45例（30%）。*EGFR*基因少见或复合突变类型与性别、年龄、分期、病理类型及吸烟史均无关。对于48例接受EGFR-TKIs治疗的患者，4组不同类型突变的患者客观缓解率（objective response rate, ORR）和疾病控制率（disease control rate, DCR）差异无统计学意义（54.5% *vs* 30.0% *vs* 0.0% *vs* 35.7%, *χ*^2^=3.200, *P*=0.34; 90.9% *vs* 85.0% *vs* 66.7% *vs* 92.9%, *χ*^2^=2.162, *P*=0.59）。中位无进展生存期（median progress free survival, mPFS）为11.0个月（95%CI: 4.4-17.6），在*EGFR*基因突变不同类型分组中分别为[15.8个月（95%CI: 9.5-22.2）、8.0个月（95%CI: 5.1-11.0）、4.9个月（95%CI: 1.4-8.4）、23.1个月（95%CI: 15.8-30.4）]（*χ*^2^=7.876, *P*=0.049）。

**结论:**

不同类型的*EGFR*少见或复合突变类型应用EGFR的疗效不尽相同，复合突变组PFS可能优于单一少见突变组。接下来有必要进一步进行大样本量的研究，发现新的敏感靶点和研究新一代的药物对于接受现有治疗效果欠佳的患者也是值得期待的。

## Abstract (en)

**Background and objective:**

Adenocarcinoma is the most common type of lung cancer. It has been clinically evaluated that therapiestargeting against the epidermal growth factor receptor (EGFR) as the clinical standard first-line treatment. The response and outcome of EGFR-tyrosine kinase inhibitors (TKIs) in patients harboring common mutations in EGFR kinase domain (deletion in exon19 and L858R in exon 21) has been well demonstrated, but not in rare or complex mutations.

**Methods:**

A total of 150 patients that harbored rare or complex mutations in EGFR diagnosed by histopathology were included in this retrospective study. The clinical-pathological characteristics of all 150 patients as well as the response and progression-free survival (PFS) in 48 patients that received EGFR-TKIs in first/second/third line treatments weredescribed and analyzed.

**Results:**

Patients were divided into four groups based on the mutation types: single G719X point mutation in exon 18 (*n*=46, 30.7%), single L861Q point mutation in exon 21 (*n*=45, 30.0%), other single rare mutation (*n*=14, 9.3%) and complex mutations (*n*=45, 30.0%). The result indicated thatthere was no correlation of *EGFR* mutation typeswith other parameters such as gender, age, clinical stage, pathology and smoking history. For the 48 patients that received EGFR-TKIs treatment, there were no significant differencesamong 4 groups in terms of objective response rate (ORR) and disease control rate (DCR) (54.5% *vs* 30.0% *vs* 0.0% *vs* 35.7%, *χ*^2^=3.200, *P*=0.34; 90.9% *vs* 85.0% *vs* 66.7% *vs* 92.9%, *χ*^2^=2.162, *P*=0.59). The median progress-free survival (mPFS) was 11.0 months (95%CI: 4.4-17.6), and in each group of different EGFR mutation types are 15.8 months (95%CI: 9.5-22.2), 8.0 months (95%CI: 5.1-11.0), 4.9 months (95%CI: 1.4-8.4) and 23.1 months (95%CI: 15.8-30.4)(*χ*^2^=7.876, *P*=0.049).

**Conclusion:**

The efficiency of targeting EGFR-TKIs on different types of rare or complex mutations was heterogeneous. The PFS may be better in patients that harbored complex mutations than those with single rare mutations. Further studies with larger sample size are necessary. Moreover, to discover novel therapeutic targets and develop new drugs are imminentfor those patientswith no response to the existing treatments.

目前，肺癌依然是全世界导致癌症相关死亡的首要原因^[1]^。肺癌可分为小细胞肺癌和非小细胞肺癌，其中非小细胞肺癌的比例达80%^[2]^。肺腺癌是非小细胞肺癌最常见的组织学类型，其比例在非小细胞肺癌中超过50%^[3]^。近年来，肺腺癌可根据其驱动基因突变类型再分为不同的临床相关的分子亚型^[4]^。特别是在非小细胞肺癌患者中发现的体细胞中的*EGFR*基因突变^[5]^。表皮生长因子受体（epidermal growth factor receptor, EGFR）是ERBB受体酪氨酸激酶家族中4个成员之一，也被称为HER1或ERBB1^[6]^。EGFR因其高度的表达，其活化可使下游信号传导增强，在各类细胞特别是在上皮细胞的增殖、存活、血管生成和迁移中起了重要作用，使其成为了非小细胞肺癌相关研究的热点^[7]^。

*EGFR*基因酪氨酸激酶结合域的突变被认为是一种积极的预后和预测指标，大多数*EGFR*突变阳性的患者对吉非替尼或厄洛替尼等酪氨酸激酶抑制剂（tyrosine kinase inhibitors, TKIs）反应良好^[8, 9]^。近年来的临床试验也表明，对于*EGFR*突变阳性的非小细胞肺癌患者，经临床验证，应用TKI类药物代替含铂的初始化疗已成为最有效的一线治疗^[10]^。19外显子框内缺失突变和21外显子L858R突变是*EGFR*敏感性突变中最主要的两种类型，占与EGFR敏感性相关的所有临床重要突变的90%^[11]^。这两种常见突变的出现与女性、不吸烟、亚裔人群以及组织学类型为肺腺癌相关^[12]^。除以上两种最常见的突变类型外，在*EGFR*基因的18-21外显子间的区域还发现了其他类型的突变^[13]^。这些少见的突变和在同一个肿瘤内同时发现的两种或更多不同类型的突变组成的复合突变目前还没有足够的研究报道，其对于TKI类药物的反应尚不完全明确^[14]^。因此本研究旨在于分析*EGFR*少见突变及复合突变类型患者的临床特征及应用EGFR-TKIs的疗效，为更好地指导少见突变及复合突变患者用药提供临床数据。

## 1 材料与方法

### 1.1 研究对象

本研究对象包含了2016年8月-2018年4月就诊于郑州大学第一附属医院的5, 147例接受了*EGFR*基因检测且为突变型的非小细胞肺癌患者中150例发生*EGFR*少见突变患者，其中48例接受了EGFR-TKIs（包括：吉非替尼、厄洛替尼、埃克替尼、阿法替尼和达克替尼）一线、二线或三线治疗至少30 d，患者治疗方案的选择并非完全根据基因检测结果，因检测结果一般在确诊后10 d-20 d方可得到。

### 1.2 基因突变的检测

所有标本均来自于组织活检或手术切除，样本均为冰冻组织或经10%福尔马林溶液固定后石蜡包块封存。基因检测采用探针扩增组织突变系统（amplification refractory mutation system, ARMS）或二代测序技术（"Next-generation" sequencing technology, NGS）进行，检测范围包括*EGFR*基因第18-21外显子。常见突变定义为*EGFR*基因19外显子缺失突变以及21外显子L858R突变；少见突变定义为*EGFR*基因除外常见突变的其他所有突变类型；复合突变定义为同一肿瘤组织标本中同时出现的2种或2种以上类型的突变。现已明确的耐药突变即*EGFR*基因20外显子T790M突变和*EGFR*基因20外显子插入突变不纳入本研究的统计和分析。

### 1.3 疗效评价

所有研究对象均于开始TKIs治疗前行计算机断层扫描（computed tomography, CT）（CT范围包含胸部、肝脏和肾上腺）、头颅磁共振成像（magnetic resonance imaging, MRI）和全身骨骼单光子发射计算机断层成像术（single-photon emission computed tomography, SPECT）评估基线水平，并于治疗30 d后进行首次复查，依照实体瘤疗效评价标准（Response Evaluation Criteria in Solid Tumors, RECIST）1.1^[15]^进行评价，评价等级分为完全缓解（complete response, CR）、部分缓解（partial response, PR）、病情稳定（stable disease, SD）和疾病进展（progressive disease, PD）。疗效评价为非PD的患者每2个月进行复查随访，可根据需要增加次数以及时发现病情变化。客观缓解率（objective response rate, ORR）定义为肿瘤缩小达到一定量并且保持一定时间的患者的比例及CR和PR占总体的比例；疾病控制率（disease control rate, DCR）定义为经治疗后获得缓解或病情稳定的患者所占评价总体的比例及CR、PR、SD占总体的比例；无进展生存期（progress free survival, PFS）定义为从EGFR-TKIs治疗开始到肿瘤进展或因任何原因死亡的时间。随访截止时间为2018年11月22日。

### 1.4 统计学分析

所有数据均使用SPSS 23.0软件进行分析处理。本研究主要分析具有少见和复合*EGFR*基因突变患者的临床特征和应用EGFR-TKIs药物的疗效及预后，分类变量采用*χ*^2^检验或*Fisher’s*精确检验；采用*Kaplan*-*Meier*法进行生存分析并行*Log*-*rank*检验；采用双侧概率检验，定义*P* < 0.05为具有统计学差异。

## 2 结果

### 2.1

少见及复合突变类型频率分布分析检出少见及复合突变患者中，单一突变共105例（70.0%），其中18外显子G719X（X=A/S/C）突变46例（30.7%），20外显子S768I突变9例（6.0%），21外显子L861Q突变45例（30.0%），19外显子插入突变4例（2.7%），18外显子E709缺失突变1例（0.7%）；复合突变共45例（30.0%），其中18外显子G719X合并20外显子S768I突变29例（19.3%），占复合突变的绝大部分，18外显子G719X合并21外显子L861Q突变4例（2.7%），18外显子G719X合并19外显子缺失突变2例（1.3%），18外显子G719X合并第3外显子R108K突变1例（0.7%），20外显子S768I合并19外显子缺失突变1例（0.7%），20外显子S768I合并21外显子L858R突变6例（4.0%），20外显子S768I合并20外显子V769L突变1例（0.7%），20外显子S768I合并18外显子G724A突变1例（0.7%）。少见及复合突变频率分布整理如表 1。

**1:** 150位患者EGFR少见及复合突变类型分布 Frequency of rare and complex *EGFR* mutations of 150 patients

Type of *EGFR* mutations	Frequency
Rare single mutations	105 (70.0%)
Exon 18 G719X (X=A/S/C)	46 (30.7%)
Exon 20 S768I	9 (6.0%)
Exon 21 L861Q	45 (30.0%)
Exon 19 insertion	4 (2.7%)
Exon 18 E709 deletion	1 (0.7%)
Rare complex mutations	45 (30.0%)
Exon 18 G719X and exon 20 S768I	29 (19.3%)
Exon 18 G719X and exon 21 L861Q	4 (2.7%)
Exon 18 G719X and exon 19 deletion	2 (1.3%)
Exon 18 G719X and exon 3 R108K	1 (0.7%)
Exon 20 S768I and exon 19 deletion	1 (0.7%)
Exon 20 S768I and exon 21 L858R	6 (4.0%)
Exon 20 S768I and exon 20 V769L	1 (0.7%)
Exon 20 S768I and exon 18 G724A	1 (0.7%)

### 2.2 患者临床病理特征

150例患者中，男性患者53例（35.3%），女性患者97例（64.7%）；中位年龄为63.5岁，范围为35岁-86岁，其中年龄＞60岁共85例（56.7%），≤60岁共65例（43.3%）；基因检测结果回示时，疾病分期为Ⅰa期-Ⅲa期的患者有14例（9.38%），Ⅲb期-Ⅳ期有81例（54.0%），55例（36.7%）患者因影像资料不全或其他原因未明确分期；147例（98.0%）患者病理诊断为肺腺癌，3例（2.0%）为非腺癌（其中有2例为肺腺鳞癌，1例为非大细胞神经内分泌癌）；94例（62.7%）患者无吸烟史，24例（16.0%）有吸烟史，32例（21.3%）患者因资料不全吸烟史不详。将患者按突变类型分为4组，分别为18外显子G719X突变46例（30.7%）、21外显子L861Q突变45例（30.0%）、其他单一少见突变14例（9.3%）和复合突变45例（30.0%）。*EGFR*基因少见或复合突变类型与性别、年龄、分期、病理类型及吸烟史均无关。患者临床病理特征整理如表 2。

**2:** 150例患者EGFR少见突变患者的临床病理特征 Clinical and pathological characteristic in 150 patients with *EGFR* rare mutations

Variable	All patients	18 G719X	21 L861Q	Other single mutations	Complex mutations	*χ*^2^	*P*
Gender							
Male	53 (35.3%)	16 (34.8%)	14 (31.1%)	6 (42.9%)	17 (37.8%)	0.822	0.84
Female	97 (64.7%)	30 (65.2%)	31 (68.9%)	8 (57.1%)	28 (62.2%)		
Age (yr)							
＞60	85 (56.7%)	22 (47.8%)	29 (64.4%)	8 (57.1%)	26 (57.8%)	2.597	0.46
≤60	65 (43.3%)	24 (52.2%)	16 (35.6%)	6 (42.9%)	19 (42.2%)		
Stage							
Ⅰa-Ⅲa	14 (9.38%)	6 (13.0%)	1 (2.2%)	2 (14.3%)	5 (11.1%)	10.179	0.10
Ⅲb-Ⅳ	81 (54.0%)	28 (60.9%)	22 (48.9%)	5 (35.7%)	26 (57.8%)		
NA	55 (36.7%)	12 (26.1%)	22 (48.9%)	7 (50.0%)	14 (31.1%)		
Pathology							
Adenocarcinoma	147 (98%)	45 (97.8%)	44 (97.8%)	14 (100.0%)	44 (97.8%)	0.769	1.00
Non-adenocarcinoma	3 (2.0%)	1 (2.2%)	1 (2.2%)	0 (0.0%)	1 (2.2%)		
Smoking history							
No	94 (62.7%)	27 (58.7%)	31 (68.9%)	7 (50.0%)	29 (64.4%)	3.037	0.81
Yes	24 (16.0%)	9 (19.6%)	6 (13.3%)	2 (14.3%)	7 (15.6%)		
NA	32 (21.3%)	10 (21.7%)	8 (17.8%)	5	9 (20.0%)		

### 2.3 应用EGFR-TKIs治疗的反应

150例少见和复合突变类型的患者中，一部分患者接受了EGFR-TKIs药物的治疗，排除无法获得治疗信息的患者，共对48例患者进行了治疗反应及PFS的分析。其中疗效评价为CR的患者为0例（0.0%），PR的患者共17例（35.4%），SD共25例（52.1%）以及PD6例（12.5%）；ORR为35.4%，DCR为87.5%。且4组不同类型突变的患者ORR和DCR差异无统计学意义（54.5% *vs* 30.0% *vs* 0.0% *vs* 35.7%, *χ*^2^=3.200, *P*=0.34; 90.9% *vs* 85.0% *vs* 66.7% *vs* 92.9%, *χ*^2^=2.162, *P*=0.59），可认为按此分组的少见和复合突变类型患者应用EGFR-TKIs药物治疗的反应即ORR和DCR无明显差异，具体见表 3。

**3:** 48例*EGFR*基因少见突变类型患者经EGFR TKIs治疗的反应 Response to EGFR TKIs treatment according to types of rare *EGFR* mutations in 48 patients

	All patients	18 G719X	21 L861Q	Other single mutations	Complex mutations	*χ*^2^	*P*
CR	0 (0.0%)	0 (0.0%)	0 (0.0%)	0 (0.0%)	0 (0.0%)		-
PR	17 (35.4%)	6 (54.5%)	6 (30.0%)	0 (0.0%)	5 (35.7%)		
SD	25 (52.1%)	4 (36.4%)	11 (55.0%)	2 (66.7%)	8 (57.1%)		
PD	6 (12.5%)	1 (9.1%)	3 (15.0%)	1 (33.3%)	1 (7.1%)		
ORR	35.4%	54.5%	30.0%	0.0%	35.7%	3.200	0.34
DCR	87.5%	90.9%	85.0%	66.7%	92.9%	2.162	0.59
CR: complete response; PR: partial response; SD: stable disease; PD: progressive disease; ORR: objective response rate; DCR: disease control rate.							

### 2.4 应用EGFR-TKIs治疗的PFS

接受EGFR-TKIs治疗的少见和复合突变患者的中位PFS为11.0个月（95%CI: 4.4-17.6），与PFS可能相关联的因素详见表 4。单因素分析可发现其中*EGFR*基因突变不同类型分组间的PFS有所不同分别为15.8个月（95%CI: 9.5-22.2）、8.0个月（95%CI: 5.1-11.0）、4.9个月（95%CI: 1.4-8.4）、23.1个月（95%CI: 15.8-30.4）（*χ*^2^=7.876, *P*=0.049），按此分组的少见和复合突变类型患者应用EGFR-TKIs药物治疗的PFS有差异，复合突变组的PFS可能优于其他单一少见突变组。

**4:** 患者经EGFR-TKIs治疗的PFS PFS in patients treated with EGFR-TKIs

	Mean (95%CI) (months)	*χ*^2^	Univariate analysis *P*
Gender		2.388	0.12
Male	16.3 (11.5-21.2)		
Female	13.9 (8.8-19.0)		
Age (year)		0.376	0.54
> 60	12.8 (8.5-17.0)		
< 60	16.8 (10.5-23.3)		
Stage		0.011	0.92
Ⅰa-Ⅲa	16.1 (0.0-32.3)		
Ⅲb-Ⅳ	16.3 (12.0-20.6)		
Smoking history		2.642	0.10
No	15.4 (10.5-20.2)		
Yes	14.0 (9.7-18.3)		
*EGFR* mutations		7.876	0.049
18 G719X	15.8 (9.5-22.2)		
21L861Q	8.0 (5.1-11.0)		
Other single mutations	4.9 (1.4-8.4)		
Complex mutations	23.1 (15.8-30.4)		
Lines of treatment		0.527	0.77
1^st^ line	14.8 (9.6-20.0)		
2^nd^ line	11.7 (8.4-15.1)		
3^rd^ line	24.4 (24.4-24.4)		
EGFR-TKIs		3.396	0.334
Gefitinib	11.5 (7.3-15.7)		
Icotinib	20.7 (13.7-27.7)		
Erlotinib	24.4		

## 3 讨论

在众多非小细胞肺癌患者中，尤其是在EGFR 19外显子缺失突变和EGFR 21外显子L858R突变的患者中，EGFR-TKIs的治疗获得了引人瞩目的疗效^[16-18]^，这两种突变占*EGFR*各类型突变的90%，因此称之为常见突变或经典突变^[11]^。既往有研究报道这两种经典突变出现在大约10%高加索人种以及50%以上亚裔人群的NSCLC中^[19]^，其应用EGFR-TKIs治疗的ORR可达60%-70%，PFS长达9个月-10个月以上，OS长达20个月以上^[20]^。

除以上两种经典突变外，少见突变如18外显子G719X突变、21外显子L861Q突变、20外显子S768I突变以及复合突变因发生频率低，目前国内外多为较小样本量的回顾性研究，一些问题尚有争论。本文对150例具有*EGFR*少见及复合突变的患者进行了临床及病理特征的分析（目前已明确的耐药突变EGFR 20外显子T790M突变和20外显子插入突变未纳入），对其中接受EGFR-TKIs治疗的48例患者进行疗效评价及生存分析，各类突变中发生频率最高者为18外显子G719X突变，与Arrieta等^[21]^报道结果一致。Yun等^[22]^的研究显示G719X突变与吉非替尼亲和力是经典型的1/50，并且研究了G719S位点突变活性是野生型的10倍。本研究中G719X单一突变占所有少见突变的30.7%，包含G719X的复合突变占24%。Wu等^[13]^的研究显示具有G719X单一突变或复合突变的患者，ORR为53.3%，中位PFS达8.1个月，本研究中G719X单一突变ORR为54.5%，中位PFS为15.8个月，含G719X复合突变ORR为30%，中位PFS为11.0个月，可见其疗效及PFS均较好，与经典突变接近。

出现频率第二位的为21外显子L861Q单一突变，占本研究少见突变的30%，另有含21外显子L861Q的复合突变占2.7%。既往研究显示此突变类型占EGFR所有突变的2%^[23]^，既往有研究报道该突变对于EGFR-TKIs敏感性低^[24]^或完全耐药^[25, 26]^。Kancha等^[26]^研究发现EGFR 21外显子L861Q突变使用吉非替尼、厄洛替尼等一代EGFR-TKIs疗效不如经典突变和18外显子G719X（S）突变，可能是因为相比于经典和18外显子G719X（S）突变来说，21外显子L861Q突变与ATP具有更高的亲和力，这导致其对ATP竞争型EGFR-TKIs的敏感性较低，而二代EGFR-TKIs的疗效更佳。本研究中，L861Q单一突变患者ORR为30%，中位PFS达8.0个月，1例为含L861Q的复合突变，中位PFS为3.2个月，疗效评价为SD，PFS与18外显子G719X突变相近。Watanabe等^[11]^的NEJ002研究中也显示，G719X和L861Q突变患者应用EGFR-TKIs疗效均逊于经典突变患者，而二者之间未见明显差异。

本研究中除了出现频率最高的18外显子G719X突变和21外显子L861Q突变外，还有一些少见单一突变如20外显子S768I突变、19外显子插入突变以及18外显子E709_T710delinsD突变。其中20外显子S768I单一突变在本研究所有少见突变中出现频率为6.0%，而包含其复合突变出现频率为25.3%，复合突变中绝大多是20外显子S768I与18外显子G719X复合突变（76.3%），Kancha等^[26]^的研究显示S768I突变对吉非替尼、厄洛替尼耐药，Chiu等^[27]^的研究显示含S768I突变应用EGFR-TKIs治疗的反应及生存时间均与G719X和L861Q接近。本研究中使用EGFR-TKIs治疗的1例S768I单一突变的患者PFS为4.0个月，疗效评价为SD，13例含S768I的复合突变中位PFS为9.73个月，ORR为38.5%。1例应用EGFR-TKIs治疗的19外显子插入突变患者，PFS为9.1个月，疗效评价为SD。1例应用EGFR-TKIs治疗的18外显子E709_T710delinsD突变应用EGFR-TKIs治疗1.7个月，疗效评价为PD。

本研究中按EGFR少见突变类型将患者分为4组，其ORR和DCR无明显差异，复合突变组的PFS可能略优于其他单一组，与Baek等^[5]^的研究结论即复合突变疗效优于单一突变一致。其解释可能是因为复合突变类型的EGFR与ATP亲和力较低，有利于EGFR-TKIs与之竞争夺得优势位置，阻止其激活下游通路引发进一步恶性增殖。在本研究中，单因素生存分析提示复合突变组PFS略优于其他单一突变组，详见图 1，但因能够分析使用的样本例数较少，未对PFS进行多因素分析，各混杂因素有待进一步平衡或消除，因此结论有待扩大样本量后进一步验证，同样因样本量较小，本文未将复合突变组中包含经典突变的类型再分组，Beak等^[5]^的研究显示复合经典突变组中位PFS为7.4个月而复合少见突变组为5.1个月，但其是否有统计学差异也有待扩大样本量后进一步研究。

**1:**
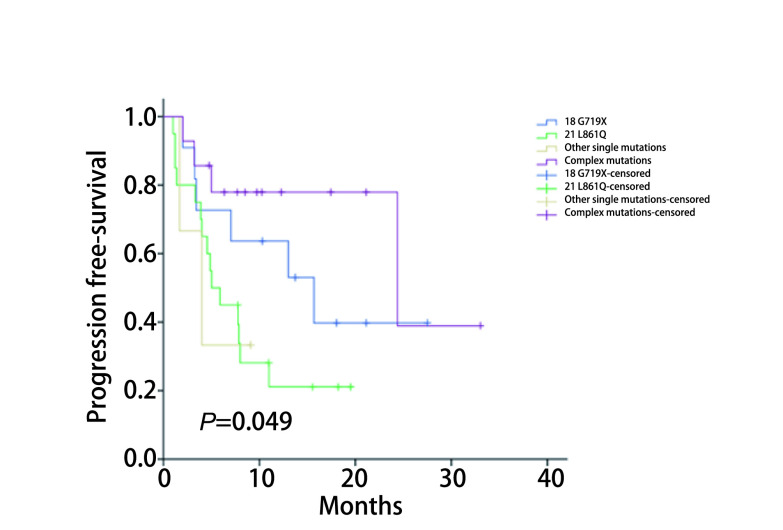
不同突变类型患者接受EGFR-TKIs治疗的PFS的*Kaplan*-*Meier*曲线 *Kaplan*-*Meier* curves showing progression free survival in response to treatment with EGFR-TKIs according to mutation status

综上所述，不同类型的EGFR少见或复合突变类型应用EGFR的疗效不尽相同，随着人们对EGFR-TKIs靶向治疗的认知逐渐加深，相关临床治疗越来越规范，急需进行大样本量的研究来更加具体地明确各类突变的特征、治疗效果及其机制，发现新的敏感靶点或者研究新的药物来针对目前EGFR-TKIs疗效欠佳的靶点都能使更多的患者从中受益。
